# Transparent deep learning to identify autism spectrum disorders (ASD) in EHR using clinical notes

**DOI:** 10.1093/jamia/ocae080

**Published:** 2024-04-16

**Authors:** Gondy Leroy, Jennifer G Andrews, Madison KeAlohi-Preece, Ajay Jaswani, Hyunju Song, Maureen Kelly Galindo, Sydney A Rice

**Affiliations:** Department of Management Information Systems, The University of Arizona, Tucson, AZ 85621, United States; Department of Pediatrics, The University of Arizona, Tucson, AZ 85621, United States; Department of Psychology, The University of Arizona, Tucson, AZ 85621, United States; Department of Management Information Systems, The University of Arizona, Tucson, AZ 85621, United States; Department of Computer Science, The University of Arizona, Tucson, AZ 85621, United States; Department of Pediatrics, The University of Arizona, Tucson, AZ 85621, United States; Department of Pediatrics, The University of Arizona, Tucson, AZ 85621, United States

**Keywords:** natural language processing, machine learning, deep learning, autism spectrum disorders, transparency

## Abstract

**Objective:**

Machine learning (ML) is increasingly employed to diagnose medical conditions, with algorithms trained to assign a single label using a black-box approach. We created an ML approach using deep learning that generates outcomes that are transparent and in line with clinical, diagnostic rules. We demonstrate our approach for autism spectrum disorders (ASD), a neurodevelopmental condition with increasing prevalence.

**Methods:**

We use unstructured data from the Centers for Disease Control and Prevention (CDC) surveillance records labeled by a CDC-trained clinician with ASD A1-3 and B1-4 criterion labels per sentence and with ASD cases labels per record using Diagnostic and Statistical Manual of Mental Disorders (DSM5) rules. One rule-based and three deep ML algorithms and six ensembles were compared and evaluated using a test set with 6773 sentences (*N* = 35 cases) set aside in advance. Criterion and case labeling were evaluated for each ML algorithm and ensemble. Case labeling outcomes were compared also with seven traditional tests.

**Results:**

Performance for criterion labeling was highest for the hybrid BiLSTM ML model. The best case labeling was achieved by an ensemble of two BiLSTM ML models using a majority vote. It achieved 100% precision (or PPV), 83% recall (or sensitivity), 100% specificity, 91% accuracy, and 0.91 F-measure. A comparison with existing diagnostic tests shows that our best ensemble was more accurate overall.

**Conclusions:**

Transparent ML is achievable even with small datasets. By focusing on intermediate steps, deep ML can provide transparent decisions. By leveraging data redundancies, ML errors at the intermediate level have a low impact on final outcomes.

## Introduction

### Background

Autism Spectrum Disorder (ASD) is a neurodevelopmental condition defined by social communication and behavioral deficits. Early detection and treatment have been shown to improve outcomes.^1^^,^^2^ The Office of Disease Prevention and Health Promotion implemented ASD-related objectives in Healthy People 2020 (MICH-29) and again in Healthy People 2030 (MICH-18) since there were few detectable changes in the proportion of children who received services before the age of four.^3^ The Centers for Disease Control and Prevention (CDC) Autism and Developmental Disabilities Monitoring Program (ADDM) shows the median age of ASD diagnosis in the United States has been unchanged since 2000 despite a dramatic increase in prevalence.^4^ National survey data shows evaluations documenting ASD but without providing an ASD diagnosis.^5^ Those without early diagnosis^6^ miss out on treatment, behavioral intervention, and community integration.^7^^,^^8^

Diagnosing ASD is time-consuming and challenging due to the heterogeneity of symptom presentation, severity, and etiology.^9^ The process requires determining the presence of symptoms within a set of diagnostic criteria. Practitioners often utilize autism-specific diagnostic tools to provide additional data. While the instruments are helpful, their sensitivity and specificity vary. A Cochrane review^10^ of the Autism Diagnostic Observation Schedule (ADOS-2), Childhood Autism Rating Scale (CARS), and Autism Diagnostic Interview-Revised (ADI-R) showed the ADOS to have the highest sensitivity with specificity being similar for all. McDonnel et al^11^ showed that using ADOS-2, clinicians were confident in only 60% of cases of an ASD diagnosis. The tools do not directly map onto the DSM criterion but focus on the generally accepted hallmark behaviors of ASD.

Researchers are working on facilitating diagnosis, for example, with systematic ASD reminders in electronic health records (EHR)^12^ or automating the process with machine learning (ML) algorithms. We focus on using EHR clinical notes to automatically identify relevant behaviors. We provide a transparent process that facilitates diagnostic assignment.

### Machine learning data for ASD case labeling

ML is used with structured data to label cases at risk for ASD. A binary label is usually assigned. In early work, a rule-based algorithm for EHR classification (*N* = 302) using Unified Medical Language concepts achieved good results (84.9% precision and 73.7% recall on the independent validation set *N* = 50) and outperformed ML algorithms such as Support Vector Machines (SVM) (66%-86.6% precision and 76.9%-85.2% recall depending on number of labels and training data combinations).^13^ Others used words with SVM with good results.^14^

Survey data with information from clinicians and parents is also used.^15^^,^^16^ Yuan et al^17^ tested several models and reported varying performance (33.2%-64.6% precision and 34.3%-91.1% recall) with a combination of notes and structured data. Interestingly, their starting point was extracting information from scanned documents containing handwritten notes. Others combine instruments, such as survey data and parental data resulting in labeling accuracy of 75.04%-85.10% (*N* = 250) depending on the age of the children and models used.^18^ Some compare ML algorithms using a public screening dataset (*N* = 292) (97%-100% precision, 100% sensitivity, and 98%-100% specificity).

Other types of data are used as well. For example, ML using brain magnetic resonance imaging scans^19^ and brain magnetoencephalography^20^ to label ASD cases showed overall lower performance across all metrics. A review of 5 years of ML research using brain imaging to diagnose ASD^21^ showed an increasing number of projects. They all suggest the existence of abnormalities, but none could replace clinical assessment. New combinations of data are also tested, eg, information tagged in home videos (*N* = 162) was combined with parental questionnaires and allowed 25% more challenging cases to be labeled.^16^. Expert-labeled behaviors in home videos (*N* = 32) were also tested with ML resulting in sensitivity ranging from 0.89% to 0.85% and specificity from 0.86% to 0.85%.^22^

### Main limitations of machine learning for ASD case labeling

Existing ML approaches have several limitations. The first is the lack of transparency, especially with deep ML, because the decisions are based on mathematical computations, not clinical knowledge.^23^ A common approach to estimate the importance of input features is to conduct ablation studies, where features are systematically withheld to estimate their effect on the outcome. Other approaches^24^^,^^25^ focus on deducing post-hoc what resulted in ML decisions, eg, focusing on layers in models such as the attention layers in transformers,^26^ or identifying important features in the best models.^27^ However, the ML features influencing the labels do not necessarily line up with clinical knowledge and may be the result of spurious correlations, eg, as seen in skin cancer detection.^28^ Further contributing to limited transparency are binary (or ternary) labels. A review of 50 projects focusing on ASD^29^ shows all projects provided a binary decision: ASD or not. While this might be sufficient for surveillance or to create triggers, binary decisions without transparency limit the opportunity for clinical judgment and potential adjustment, leading to a lower applied utility.

A second limitation of ML is the need for large data sets that can serve as gold standards.^30^ To create a large amount of data, EHRs are used because they are accessible due to the diagnostic code associated with each encounter. However, developing and tweaking algorithms using larger datasets and complex models does little to improve the diagnostic process.

A final limitation comprises problems encountered with unbalanced data. The amount of information in the records as well as the number of cases in a set can dramatically change the outcome of algorithms. Even with large sets of records, the number of ASD cases may be small, resulting in few training examples. Several techniques can be applied, such as collecting more data, over- and under-sampling of existing data,^31^ creating synthetic data, eg, with large language models (LLM),^32^ or applying techniques such as the Synthetic Minority Over Sampling Technique (SMOTE).^33^

## Methods

### Overview

We propose a transparent ML decision process by focusing on intermediate steps and using clinical rules for a final label. This is different from explainable AI (XAI) approaches such as SHAP^24^ or LIME^25^ where explanations are added post-ML decision. Our transparency comes from using ML for individual behaviors that map to the DSM (Table 1) before a decision and that are combined using DSM5 rules. We were among the first to assign criterion labels using DSM-IV^34–37^ and have adapted to DSM5. For case labeling, we use the CDC guidelines for DSM5^38^: we label a case as ASD when there are examples of three A criteria and at least two B criteria as defined in the DSM5. We do not take the number of examples for each criterion into account as this is also not done in clinical practice. For example, there may be 10 examples of A1, but it suffices if one A1 example is correctly labeled to contribute to the final case label.

**Table 1.** Example DSM5 criteria for ASD (Full criteria list is available online^39^).

Criterion	DSM description (extract)	EHR example
A1	Deficits in social-emotional reciprocity, …	He appeared fairly indifferent to his peers and did not initiate any interactions.
		Both parent and teacher rate him as having poor pragmatic language skills.
A2	Deficits in nonverbal communicative behaviors used for social interaction, …	Does not use appropriate social communication eye contact, etc..
		Does not make eye contact.
A3	Deficits in developing, maintaining, and understanding relationships, …	During free play, he played by himself with blocks and miniature replications of road signs.
		At home, mother reports he prefers to play by himself.
B1	Stereotyped or repetitive motor movements …	Mother’s concerns include that she seems to repeat word a lot and will say the same sentence over and over and over again.
		She repeats phrases that are said. She is prone to engage in repetitive activities.
B2	Insistence on sameness	Both at home and school, he becomes very upset if the routine is changed.
		His mother reports that he has difficulty adapting to changing situations and that he takes longer to recover from difficult situations than most others his age.
B3	Highly restricted, fixated interests that are abnormal in intensity or focus	had some difficulty interrupting this pattern once it started and even though she clearly enjoys joint play she became fixated on her simple back and forth movement with the objects.
		She plays with the same things over and over she is described to be fixated on superheroes
B4	Hyper- or hyporeactivity to sensory input	He was observed to be sensitive to unexpected noises by covering his ears.
		He demonstrates stereotypical movements, irregularities and impairments in communication, and unusual responses to sensory experiences.

### Data set

We used the Arizona ADDM data. The data comprises information on individuals with autism, intellectual disability, and other related conditions including evaluations performed with verbatim text of results and clinical impressions as well as data for autism-specific tests. We started with ADDM records^40^ using DSM-IV.^41^ In 2013, the DSM was updated to DSM5 and the diagnostic criteria changed.^42^ The Arizona ADDM reviewer (CDC-trained) annotated our dataset using the new labeling approach. She included phrase mapping onto the specific DSM5 criteria and added the diagnostic label using DSM5 rules. Currently, no sentence-level labels are retained by the CDC, making the acquisition of new datasets futile. Because relabeling all records was cost-prohibitive, we had to decide to use either a smaller, but current dataset (DSM5), or a larger but outdated dataset (DSM-IV). We chose to use a smaller dataset. The ADDM’s reviewers' interrater reliability is very high for every diagnostic criterion^43^ allowing us to rely on a single expert.

The annotator relabeled 185 records collected in 2014 and 2016 for which she had served as the original reviewer. We used 150 records for training and 35 records for testing (Table 2). There were 18 among the 35 that received an ASD diagnosis (51.4% majority baseline). All records are of children who exhibit ASD-like behaviors and 51% received an ASD diagnosis. As such, the cases without ASD labels are not extremely different from others making this a challenging ML task.

**Table 2.** Dataset description.

Counts	Training set	Test set
	(*N*= 150)	(*N* = 35)
Sentences	34 313	6773
A1 labeled sentences	855	224
A2 labeled sentences	471	109
A3 labeled sentences	524	143
B1 labeled sentences	539	113
B2 labeled sentences	338	105
B3 labeled sentences	146	69
B4 labeled sentences	697	151

We elected to set aside a representative test set instead of 10-fold cross-validation to avoid contaminating the test set for the evaluation of our rule-based parser. Since human effort is involved in creating rules, an unseen test set is required for the evaluation. With multiple test sets, the human developers would have to “unsee” information when creating rules for different folds. In addition, no ML training or tuning was done using this test set. This is common when rule-based approaches are used, eg, with common challenges.^44^^,^^45^ In addition, with this set, we can ensure clinical relevance. Testing on additional folds would show unrealistically high and low performance and would not contribute in terms of external validity. Others have also used a train/tests loop instead of n-fold cross-validation, eg, to balance datasets in the test set^13^ or to test on a new real-world dataset.^18^ The train/tune/test is also used with several machine learning where tuning is limited to one step.^46^

Since our algorithms are trained at the sentence level, our training set contains 34 313 sentences, and our test set 6773 sentences. We do not use any structured data or outcomes from screening instruments. Although our dataset is imbalanced, we did not use oversampling since it does not affect the rule-based parser but would create unequal conditions for the algorithm comparison. Furthermore, we aim to discover the virtue of using ensembles based on the same datasets.

### Algorithms

**Figure 1.**
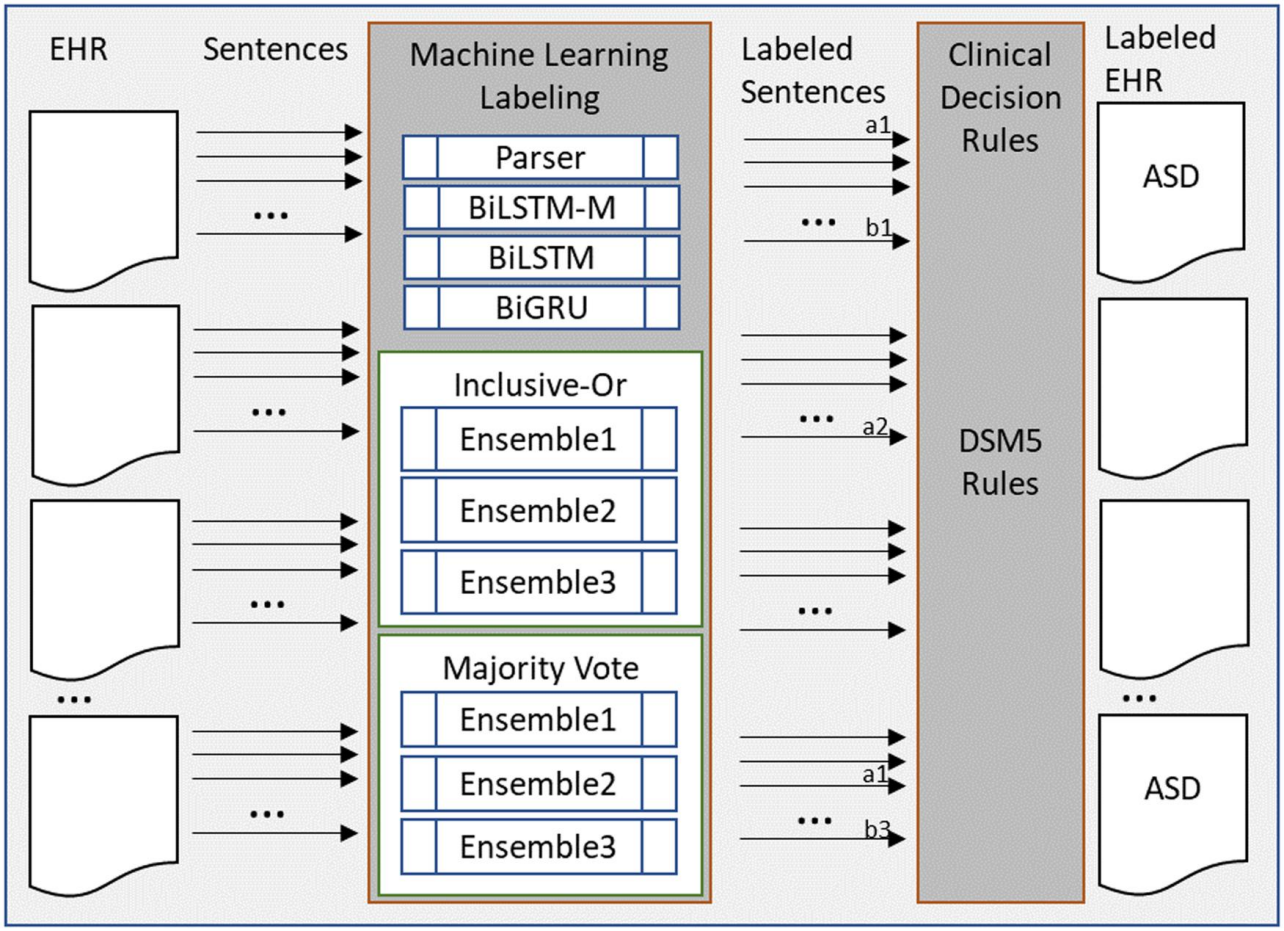
Overview of algorithm combinations.

Our goal is to combine algorithms that focus on intermediate steps important for a diagnosis. We include a rule-based and three deep MLs selected after a variety of versions and settings were tested. These individual components are consolidated into two types of ensembles (Figure 1).

The rule-based parser was created for the autism criteria in the DSM-IV but adjusted for the DSM5 criteria. New rules were created in two phases each analyzing twenty annotated EHRs at a time. The current DSM5 parser leverages 193 rules and 304 lexicons. The patterns identify different behaviors matching DSM5 criteria (A1-A3, B1-B4).

ML models were developed in Python (3.7) using Tensorflow (2.x). A BiGRU (Bidirectional Gated Recurrent Unit) was trained separately for each criterion label (A1-A3, B1-B4). In pilot work, we evaluated training this model for multi-labeling, ie, the algorithm is trained to assign all labels, but the performance was lower. The model used here was trained for two epochs (128 GRUs, 9 layers, batch size = 1000) and uses Global Vectors (GloVe)^47^ pre-trained embeddings (300 dimensions, 6 billion tokens, and a 400k vocabulary).

We also trained an ensemble Bidirectional Long Short-term Memory (BiLSTM to identify criterion labels (A1-A3, B1-B4)). We incorporate expert knowledge into the BiLSTM model by adding information from the rule-based parser. Terms matching the parser lexicons were concatenated to the input. We found a small boost in performance with this additional information over a BiLSTM using only embeddings. The best model was trained for 10 epochs (batch size = 16, Bidirectional units = 150, parser input units = 10, attention layer units = 20, final layer units = 150)) and using GloVe^47^ embeddings (300 dimensions).

Finally, we trained a multilabel BiLSTM (BiLSTM-M) neural network without parser input. After testing different models, the multilabel version performed best. The model selects each label that has a predicted value (in the model) above a threshold (set at 0.5). Other parameters were retained from the LSTM above.

The individual models are combined in ensembles. Each algorithm represents knowledge differently and allows ensembles to leverage the differences. The output of the models comprises the sentences from the EHR (with unique identifiers), and the DSM label assigned. We compared six ensembles. Our first ensemble combines all algorithms, the second combines the ML models (ie, no parser), and the third combines the two top-performing ML algorithms. Each ensemble uses two approaches to combine output. The first is a “majority vote” which assigns a label to a sentence if the majority of the models assign this label to a sentence. This is a strict model where multiple individual models need to agree on the label. Our second approach uses “inclusive or” where a sentence is assigned a label if any of the algorithms assign the label. This is less restrictive and will assign a label to a sentence if any of the models assigns this label.

### Metrics

We calculate five measures of model performance defined using true positive (TP), true negative (TN), false positive (FP), and false negative (FN), actual positive (P), actual negative (N), predicted positive (PP), and predicted negative (PN) counts.

Precision (positive predictive value) and recall (sensitivity) are commonly used in both ML and medicine. F1 is the harmonic mean of precision and recall and indicates how well-balanced a system is. The F1 value will be low if either precision or recall is low. These are the most informative for our sentence labeling.

Accuracy is a typical ML metric indicating how well a decision is made out of all decisions that ML made. This measure is especially important for case labeling since it shows how much better an algorithm performs against the majority baseline, ie, assigning the most common label (51% in our test set since there are 18 ASD cases among 35). Finally, we also include specificity as a typical metric in medicine.

- Accuracy = (TP + TN)/(P + N)
- Precision or Positive Predictive Value (PPV) = TP/PP
- Recall or Sensitivity = TP/P
- F1 = 2 × TP/(2 × TP + FP + FN)
- Specificity = TN/N

The best-performing ML model was also compared with autism-specific diagnostic tests available in ADDM data using chi-squared analysis.

## Results

### Criterion labeling

We summarize performance results. Details are shown in Table S1.

When averaging over all 7 different diagnostic criteria, average precision was the highest for the multilabel LSTM (67%), average recall was the highest for the hybrid LSTM (52%), and the multilabel LSTM achieved the highest F-measure (0.57). The parser scores higher on precision (49%) than on recall (35%) for individual criteria and scored lower than the ML models. The BiGRU shows 58% precision and 47% recall, the hybrid LSTM is more balanced with 54% precision and 52% recall, and the multilabel LSTM scores 67% precision and 51% recall.

The BiGRU was unable to label any sentences for the B3 criteria, resulting in a score of 0 for each measure for B3 (hereby lowering average precision and recall). Most models performed worse for this criterion, and it is the criterion for which the fewest examples are available. All models performed well for the A2 criteria even though this is not the criterion with the most examples available.

Table 3 shows the results for the ensembles. Results for combining algorithms using inclusive or showed the highest F-measure (0.58) when the top ML models were combined. This combination also led to the highest precision (61%). However, the highest recall (70%) was found when the four algorithms were combined. The same combinations were tested with a majority vote. The highest F-measure (0.58) was found when all ML algorithms were combined. However, the highest precision (76%) was found when all four algorithms were combined, and the highest recall (51%) when the ML algorithms were combined. When comparing the two types of ensembles, the majority vote ensemble led to higher precision and the inclusive or ensembles show higher recall.

**Table 3.** Criterion label results for the ensembles (*P* = Precision or PPV, R= Recall or Sensitivity, F1 = Harmonic Mean of Precision and Recall).

	All four algorithms			All ML algorithms			Top two algorithms		
	P	R	F1	P	R	F1	P	R	F1
**Inclusive or ensembles**									
**A1**	0.38	0.74	0.50	0.42	0.71	0.53	0.54	0.61	0.57
**A2**	0.52	0.78	0.62	0.58	0.73	0.65	0.69	0.50	0.58
**A3**	0.41	0.61	0.49	0.55	0.57	0.56	0.64	0.52	0.58
**B1**	0.42	0.79	0.55	0.50	0.73	0.59	0.61	0.68	0.64
**B2**	0.46	0.64	0.54	0.55	0.60	0.58	0.71	0.53	0.61
**B3**	0.46	0.57	0.51	0.48	0.52	0.50	0.59	0.39	0.47
**B4**	0.40	0.81	0.53	0.44	0.79	0.57	0.47	0.77	0.58
Avg.	0.44	0.70	0.54	0.50	0.67	0.57	0.61	0.57	0.58
**Majority vote ensembles**									
**A1**	0.76	0.40	0.53	0.63	0.54	0.58	0.78	0.39	0.52
**A2**	0.98	0.54	0.70	0.78	0.60	0.68	0.95	0.50	0.66
**A3**	0.81	0.36	0.50	0.75	0.45	0.57	0.80	0.38	0.52
**B1**	0.73	0.52	0.61	0.71	0.58	0.64	0.82	0.52	0.64
**B2**	0.82	0.39	0.53	0.79	0.46	0.58	0.82	0.40	0.54
**B3**	0.56	0.07	0.13	0.66	0.28	0.39	0.00	0.00	0.00
B4	0.67	0.56	0.61	0.60	0.69	0.64	0.64	0.60	0.62
Avg.	0.76	0.41	0.51	0.70	0.51	0.58	0.69	0.40	0.50

Some errors were clearly false positives or false negatives. Other behaviors required subtle reading of the entire record for a label. The following examples are from evaluating sentences labeled with A1 by the inclusive or ensemble:

- TP: “OT: He does not answer to his name being called.”, “He imitates gross motor movements, but did not attempt to imitate facial expressions.”
- FP: “School living: He does not show respect for the property and rights of other people or offer to help.”, “He did not engage in adult directed interactions but would engage with free play.”
- FN: “Teacher indicated that he often bothers other children, does not take turns and is unable to explain personal experiences.”, “He is beginning to verbally interact with peers while playing at centers.”
- TN: “ARCHE Autism Evaluation.”, “Reason for Referral: Child attended a preschool screening and was referred for an evaluation in the areas of cognition, communication and social-emotional skills.”

### Case labeling

Based on the criterion labels, each case was assigned a label for ASD or no ASD. Detailed results are available in the Table S2.

Overall, the best performance was achieved with the ensemble containing the two top ML algorithms (BiGRU and multilabel LSTM) which achieved 100% precision, 83% recall (or sensitivity), and 100% specificity. Its accuracy of 91% is much higher than the majority baselines of 51.4% (18 out of 35 cases labeled ASD). Among the single algorithms, the BiGRU by itself achieved the highest performance, with 89% for each measure except 88% for specificity.

**Figure 2.**
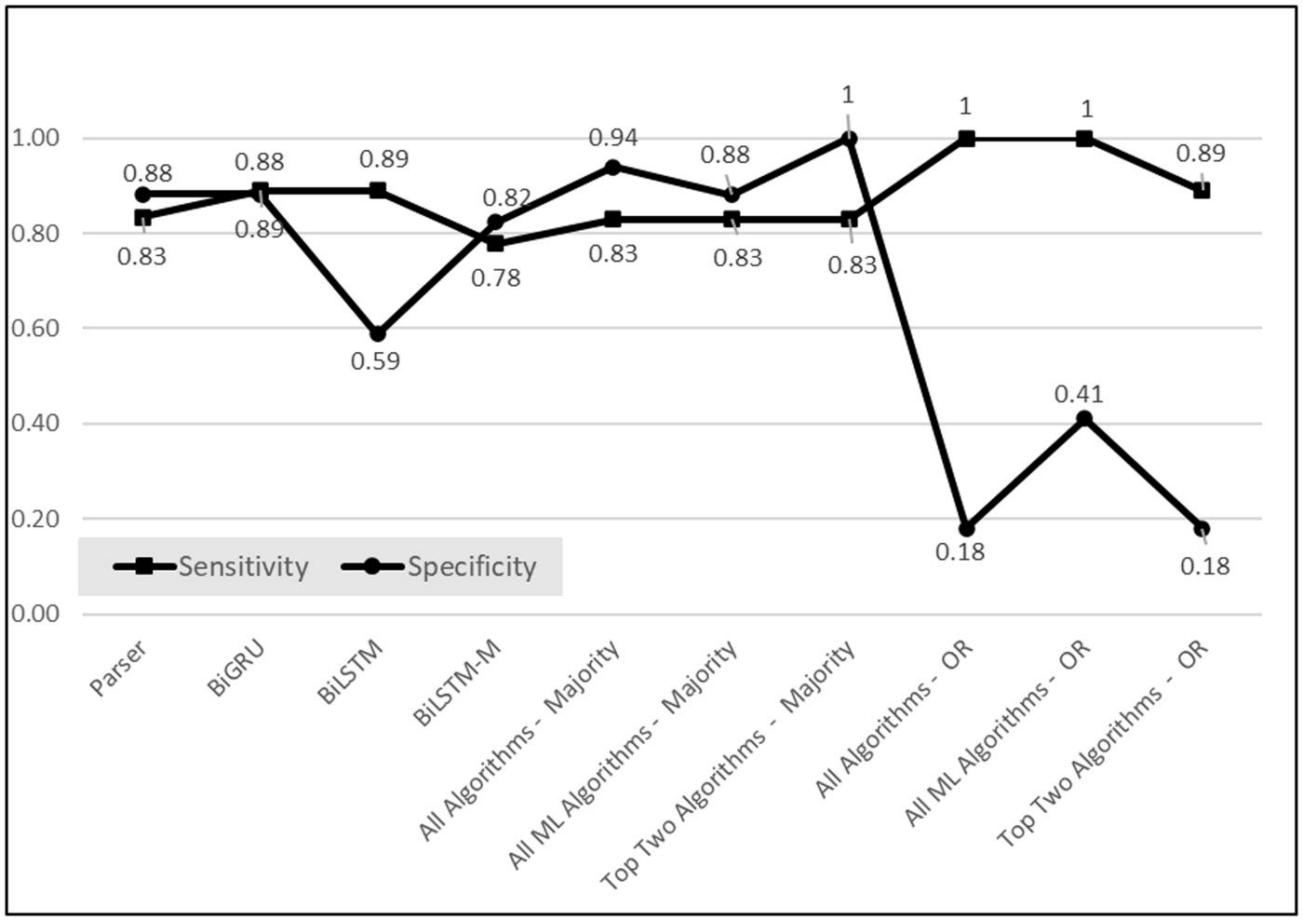
Sensitivity and specificity of case labeling.

Figure 2 shows the superiority of combining algorithms using the majority vote for specificity with much lower specificity when an inclusive or is used. Most algorithms performed well on sensitivity.

### Case labeling instrument comparison

Our original data set includes results for autism-specific diagnostic instruments when administered. We compare the results of our approach with the decision that could be made using these diagnostic instruments.

Of our 35 cases, there were 30 where at least one diagnostic instrument was applied and 14 wherein actual results were stated. We used the test results to assign a label as follows:

- Asperger Syndrome Diagnostic Scale^48^ (ASDS) scores >110 as ASD, scores between 90-110 inclusive as Probable ASD, all else not ASD.
- Childhood Autism Rating Scale^49^ (CARS) all results listed as ASD (Severe, Mild to Moderate, NOS) as ASD, all else not ASD.
- Gilliam Asperger Disorder Scale^50^ (GADS) scores High/Probable as ASD, scores listed as Borderline as Probable ASD, all else not ASD.
- Gilliam Autism Rating Scale^51^ (GARS) scores >110 as ASD, scores between 80-110 inclusive as Probable ASD, all else not ASD.
- GARS2 scores ≥85 as ASD, all else not ASD.
- Modified Checklist for Autism in Toddlers^52^ (MCHAT) scores ≥2 as ASD, all else not ASD.
- Autism Diagnostic Observation Schedule^53^^,^^54^ (ADOS) is scored the same as our ML models—ASD or Not ASD.

There were cases with duplicate testing that demonstrated conflicting results. One individual had two GADS tests at the same age with one scoring as No ASD and another as borderline ASD. The other individual had 4 tests, a GARS2 around age 5 which was scored as ASD, then 2 CARS and one ASDS around age 9 which all scored as No ASD. Both individuals have ASD based on the gold standard. All tests were included since we are assessing the ability of our algorithms and existing autism tests in comparison with CDC reviewer decisions.

When any diagnostic test indicated autism, sensitivity was low and specificity high. Our majority vote ensemble performed with higher sensitivity (0.83) and specificity (1.0) than the best diagnostic tests (Table S3).

Table 4 provides chi-square test results for independence results for the ASD diagnostic tests and our best ensemble for the expected counts of ASD and No ASD. There was no significance for the Diagnostic Test group whereas our best ensemble was much more likely to rule out ASD. (*P* = .01, standardized residuals 2.32).

**Table 4.** Chi-Square comparisons of predicted cases by ensemble and ASD tests.

	Gold standard label		
	ASD	No ASD	
	*N* = 11	*N* = 3	*P*-value
**Diagnostic tests**			1.00
ASD	7 (63.6%)	2 (66.7%)	
No ASD	4 (36.4%)	1 (33.3%)	
**Majority vote Ens. of top 2 algorithms**			.011
ASD	10 (90.9%)	0 (0.00%)	
No ASD	1 (9.09%)	3 (100%)	

## Discussion

### Principal results

Today, ML researchers are turning towards increasingly complex models. Our work shows that by leveraging domain knowledge, other avenues beyond brute-force computation may perform well. Our main contributions are twofold.

First, we showed how small datasets can serve well by focusing on intermediate steps and leveraging data redundancy. We evaluated individual and ensemble ML models for their ability to assign all intermediate criteria labels. However, not every example needs to be labeled correctly to assign a diagnostic label. For example, with an imperfect recall of criteria, a correct final case decision can be made when enough (but not necessarily all) correct examples are found. Similarly, with imperfect precision, when enough examples are identified correctly (even when others are in error) this allows for a correct case decision to be made. As a result, our ML algorithms provide sufficient information when combined. These results are better than those of others using similar ADDM records. For example, Maenner et al^55^ used ADDM records with random forest ML and 175 preselected phrases from the notes to classify records as ASD with 89% precision and 84% recall. Ensembles achieve better results and show how stricter labeling (majority vote) results in higher precision but lower recall, and vice versa for more lenient labeling (inclusive or), as expected. Overall, our best ensemble classified cases more accurately as ASD or No ASD than the clinical autism tests present in the case records.

**Figure 3.**
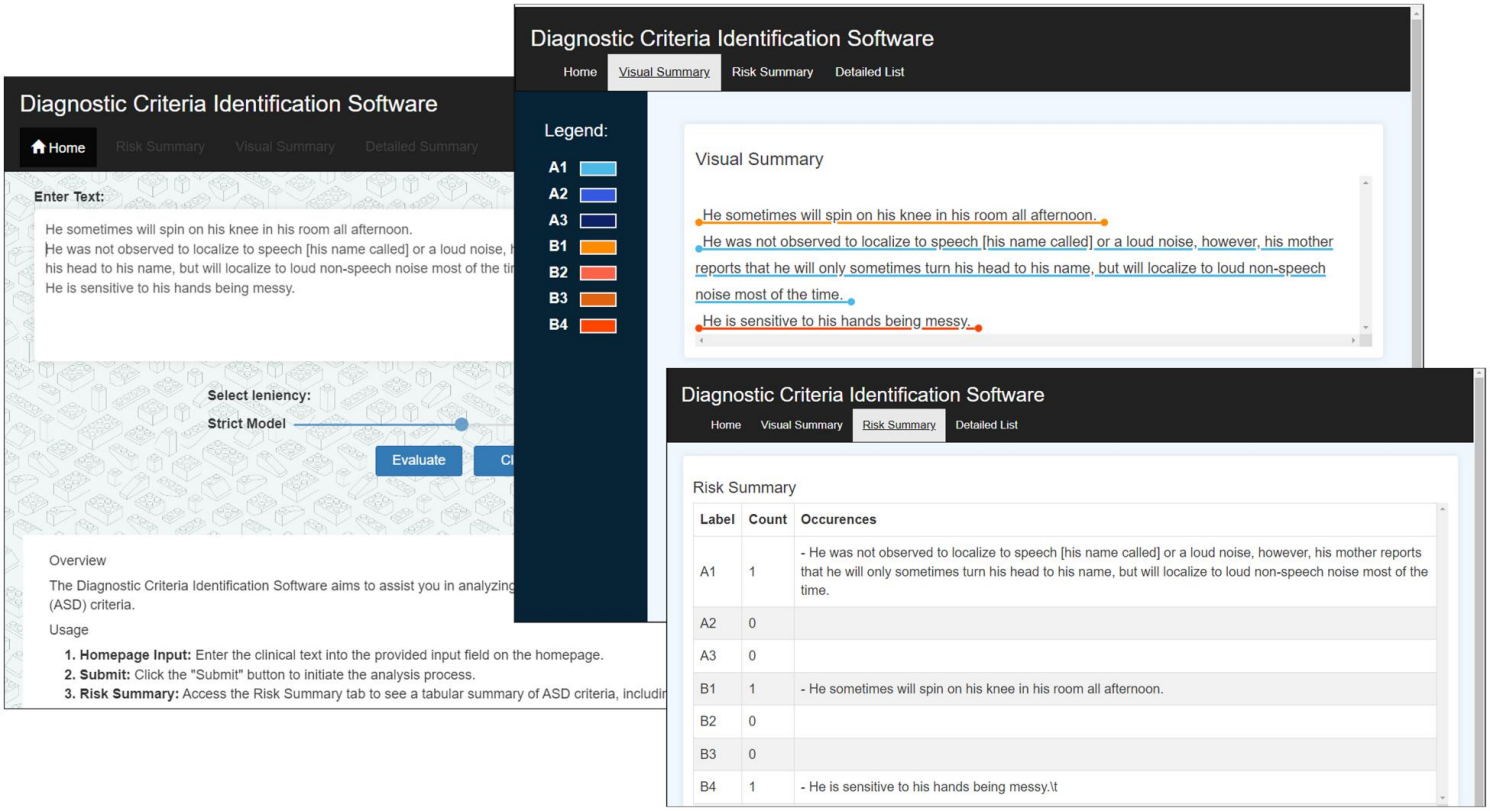
Early prototype to present ML model output.

Our second contribution is that our approach strives to bring transparency. First, almost all other work provides only a binary label. An exception is the work by Thabtah and Peebles^56^ who compared interpretable rules from ML. Their algorithms produced in most cases over 20 rules and slightly lower performance in comparison to standard ML. In contrast, our process allows clinicians to apply clinical judgment in interpreting the criterion label assignment and the final case label. Figure 3 shows our online prototype.

In addition, we also believe our approach may help avoid spurious correlations. Some sentences, eg, “ARCHE Autism Evaluation”, might be correlated with an ASD diagnosis and could be used incorrectly by black-box models. However, such sentences are never labeled as relevant behavior by any of our models and would also easily be recognized as irrelevant by a human.

### Limitations

There are several limitations to our work. First, our work occurs in a low-resource domain where it is expensive and time-consuming to create additional training data. Using unstructured EHR data requires clinical content knowledge and an understanding of issues that can arise in secondary analysis of clinical datasets. We will investigate the use of synthetic data to increase our training data.

The second limitation is that we used high-quality and text-rich records for our work. Traditional EHR records will contain less information and the language used in primary care differs significantly. We expect lower performance in subsequent model performance and the need to tune the ML models for new EHR. This is a common problem, and several potential solutions can be tested. In addition, clinical settings will differ, and further testing will be needed to evaluate our approach when different levels of prevalence are observed and for the ability to support differential diagnosis.

The third limitation is that limited resources were used. Longer training times, more algorithms, and more ensembles will be tested. In addition, different word embeddings will be tested, including larger existing resources such as Bio-BERT as well as in-house developed ASD-specific embeddings.^57^ Furthermore, this work did not apply n-fold cross-validation. This was a necessary trade-off since it would artificially lower scores. Some labels were rare and would not be learned due to limited presence in the training data folds. With more data, the standard n-fold cross-validation can be applied.

The fourth limitation is that we only focused on information in the narrative. Our EHR data contain additional structured information, such as demographic information and other clinical conditions. Such additional information may increase performance. Collecting and combining more and new data, eg, biomarkers^58^ or information from videos,^16^ may significantly enhance results and still retain transparency.

Finally, our algorithms show excellent results for criteria and case labeling. Since our work is based on one dataset, some overfitting for this dataset may have increased performance. In addition, gold standard labels were assigned by one expert. Such gold standards are sometimes easier to label than those created by a team since a team approach may increase variation when compromises are needed in the decision.^59^

## Conclusions

In this project, we provide evidence that applying ML models to small datasets can yield good results when redundant information is present. Focusing ML on intermediate, clinical steps and not the final label can provide transparency and possibly avoid spurious correlations. We believe our approach will provide valuable support to clinicians during the ASD diagnostic process. In future work, we will increase our data sets, types of data, and training time, transfer to different EHRs, and develop a transparent decision support system for clinicians.

## Supplementary Material

ocae080_Supplementary_Data

## Data Availability

The EHR data used to train the ML algorithms and to evaluate the diagnostic tests are not publicly available due to the ADDM Network Data Confidentiality and Security Agreement. The parameters used to train the ML algorithm are described in the manuscript. The ML algorithms themselves are available through open-source libraries as listed in the manuscript.
