# Supplementary material for: Transparent deep learning to identify autism spectrum disorders (ASD) in EHR using clinical notes
Source: J Am Med Inform Assoc. 2024 Apr 16;31(6):1313–21. doi: 10.1093/jamia/ocae080 (PMC11105145; doi:10.1093/jamia/ocae080)
Supplement: ocae080_Supplementary_Data [file ocae080_supplementary_data.docx]

**Supplementary Materials**

Table 1: Criterion label results for the individual algorithms (P = Precision or PPV, R= Recall or Sensitivity, F1 = Harmonic Mean of Precision and Recall)

|  | **Parser** | | | | **BiGRU** | | | **BiLSTM** | | | **BiLSTM-M** | | | |
| --- | --- | --- | --- | --- | --- | --- | --- | --- | --- | --- | --- | --- | --- | --- |
|  | | **P** | **R** | **F1** | **P** | **R** | **F1** | **P** | **R** | **F1** | | **P** | **R** | **F1** |
| **A1** | | 0.47 | 0.29 | 0.36 | 0.71 | 0.46 | 0.56 | 0.43 | 0.58 | 0.49 | | 0.55 | 0.54 | 0.54 |
| **A2** | | 0.69 | 0.47 | 0.56 | 0.70 | 0.55 | 0.62 | 0.61 | 0.61 | 0.62 | | 0.89 | 0.59 | 0.71 |
| **A3** | | 0.45 | 0.42 | 0.43 | 0.74 | 0.48 | 0.58 | 0.60 | 0.41 | 0.48 | | 0.66 | 0.43 | 0.52 |
| **B1** | | 0.45 | 0.53 | 0.49 | 0.61 | 0.63 | 0.62 | 0.53 | 0.56 | 0.54 | | 0.76 | 0.53 | 0.62 |
| **B2** | | 0.51 | 0.35 | 0.42 | 0.80 | 0.46 | 0.58 | 0.54 | 0.44 | 0.48 | | 0.71 | 0.48 | 0.57 |
| **B3** | | 0.52 | 0.17 | 0.26 | 0.00 | 0.00 | 0.00 | 0.48 | 0.41 | 0.44 | | 0.59 | 0.39 | 0.47 |
| **B4** | | 0.34 | 0.24 | 0.28 | 0.52 | 0.74 | 0.61 | 0.56 | 0.61 | 0.59 | | 0.56 | 0.63 | 0.59 |
| **Avg.** | | **0.49** | **0.35** | **0.40** | **0.58** | **0.47** | **0.51** | **0.54** | **0.52** | **0.52** | | **0.67** | **0.51** | **0.57** |

Table 2: Case label results for individual algorithms and ensembles (P = Precision or PPV, R= Recall or Sensitivity, F1 = Harmonic Mean of Precision and Recall)

| **N = 35** | **P** | **R** | **F1** | **Specificity** | **Accuracy** |
| --- | --- | --- | --- | --- | --- |
| **Individual algorithms:** |  |  |  |  |  |
| Parser | 0.88 | 0.83 | 0.86 | 0.88 | 0.86 |
| BiGRU | 0.89 | 0.89 | 0.89 | 0.88 | 0.89 |
| BiLSTM | 0.70 | 0.89 | 0.78 | 0.59 | 0.74 |
| BiLSTM-M | 0.82 | 0.78 | 0.80 | 0.82 | 0.80 |
| **Ensemble – All Algorithms:** |  |  |  |  |  |
| Or Ensemble | 0.56 | 1 | 0.72 | 0.18 | 0.60 |
| Majority Vote Ensemble | 0.94 | 0.83 | 0.88 | 0.94 | 0.89 |
| **Ensemble – All ML Algorithms:** |  |  |  |  |  |
| Or Ensemble | 0.64 | 1 | 0.78 | 0.41 | 0.60 |
| Majority Vote Ensemble | 0.88 | 0.83 | 0.86 | 0.88 | 0.89 |
| **Ensemble – Top Two Algorithms:** |  |  |  |  |  |
| Or Ensemble | 0.84 | 0.89 | 0.86 | 0.82 | 0.86 |
| Majority Vote Ensemble | 1 | 0.83 | 0.91 | 1 | 0.91 |

Table 3: Comparison in diagnostic labeling with different instruments

|  |  | **Gold Standard Label** | |  |  |
| --- | --- | --- | --- | --- | --- |
|  |  | **ASD** | **No ASD** | **Sensitivity** | **Specificity** |
| Any Diagnostic Test (N= 14) | | | | 0.20 | 0.78 |
|  | ASD | 7 | 2 |  |  |
|  | No ASD | 4 | 1 |  |  |
| CARS (N= 4) | |  |  | 0.00 | 1.00 |
|  | ASD | 2 | 0 |  |  |
|  | No ASD | 2 | 0 |  |  |
| MCHAT (N= 4) | |  |  | NA | 0.5 |
|  | ASD | 2 | 2 |  |  |
|  | No ASD | 0 | 0 |  |  |
|  |  |  |  |  |  |
|  |  |  |  |  |  |
| Majority Vote Ens. of Top 2 Algorithms (N=35) | | | | 0.83 | 1.0 |
|  | ASD | 15 | 0 |  |  |
|  | No ASD | 3 | 17 |  |  |
